# Considerations in cultural adaptation of parent–child interventions for African American mothers and children exposed to intimate partner violence

**DOI:** 10.3389/fpsyg.2024.1295202

**Published:** 2024-04-23

**Authors:** Breana R. Cervantes, Madeleine Allman, Quenette L. Walton, Ernest N. Jouriles, Carla Sharp

**Affiliations:** ^1^Department of Psychology, University of Houston, Houston, TX, United States; ^2^Graduate College of Social Work, University of Houston, Houston, TX, United States; ^3^Department of Psychology, Southern Methodist University, Dallas, TX, United States

**Keywords:** African American families, parent–child intervention, intimate partner violence, cultural adaptation, parenting

## Abstract

African American women are at disproportionate risk of experiencing intimate partner violence (IPV) and consistently report more severe and recurrent IPV victimization in comparison to their White and Hispanic counterparts. IPV is more likely to occur in families with children than in couples without children. Parenting in the wake of IPV is a challenging reality faced by many African American women in the United States. Despite the urgent need to support mothers who have survived IPV, there is currently no culturally adapted parenting intervention for African American mothers following exposure to IPV. The aim of this review is to summarize and integrate two disparate literatures, hitherto unintegrated; namely the literature base on parenting interventions for women and children exposed to IPV and the literature base on parenting interventions through the lens of African American racial and cultural factors. Our review identified 7 questions that researchers may consider in adapting IPV parenting interventions for African American women and children. These questions are discussed as a possible roadmap for the adaptation of more culturally sensitive IPV parenting programs.

## Introduction

Intimate partner violence (IPV), defined as physical and/or sexual violence, stalking, and/or psychological aggression inflicted by a current or former intimate partner (Breiding et al., 2015), is a significant public health concern that leads to harmful short-term and long-term physical and mental health consequences, particularly for women (e.g., Cimino et al., 2019). Nearly one in three women in the United States experience stalking and/or physical or sexual IPV at least once in their lifetime (Basile et al., 2011; Smith et al., 2018). While 41.2% of African American women experience physical victimization by an intimate partner in their lifetime, women overall experience lower rates (31.5%) (DuMonthier et al., 2017). Further, in 2020, African American women were victims of intimate partner homicide at a rate nearly three times higher than their White counterparts (DuMonthier et al., 2017; Violence Policy Center, 2022).

African American women experience IPV in qualitatively different ways than women from other racial groups and thus, are disproportionately impacted by IPV (Powell, 2008). For instance, African American women encounter systemic and institutional racism, historical trauma, and the resultant structural inequalities, which elevate the risk of IPV, perpetuate domestic violence and create barriers to formally reporting or seeking help for IPV (West, 2012; Kelly et al., 2020). Moreover, African American women encounter additional challenges in the wake of experiencing IPV, such as revictimization by the criminal justice system, increased risk of losing custody of their children, and inadequate healthcare (DuMonthier et al., 2017). Indeed, their lived experiences of racism, discrimination, and neglect from the intersecting criminal justice and health care systems and anticipation of an adverse experience with health providers and law enforcement deter them from seeking help and utilizing available domestic violence services until their abuse is at “peak lethality level” (Waller et al., 2022; Waller and Bent-Goodley, 2023).

The COVID-19 pandemic and resultant stay-at-home orders have exacerbated IPV issues over the last three years, with rates of IPV rising due to economic instability, heightened stress, increased opportunity for relational conflict, and the breadth of IPV resources (e.g., hotlines, shelters) becoming narrower due to limited shelter availability and police efforts to limit arrests to felonies (Buttell and Ferreira, 2020; Evans et al., 2020). Recent findings on the impact of COVID-19 on IPV survivors underscores the disproportionate risk for IPV for African American women, which is consistent with a “dual pandemic” perspective on African American women’s heightened vulnerability to COVID-19 and IPV due to notable social determinants of health, social injustice, and racism (Wood et al., 2023). Against this background and the recently increased salience of IPV related to COVID-19, effective interventions are critical to addressing this serious public health issue among African American women.

### Impact of IPV exposure on child outcomes and caregiving behaviors

Many women who are impacted by IPV are also mothers. Research on the associations between exposure to IPV and child outcomes consistently concludes that children who are exposed to IPV suffer a variety of harmful consequences that impact their psychological, emotional, behavioral, cognitive, and physical well-being and span from infancy through late adolescence (Carlson et al., 2019). Indeed, some authors consider IPV exposure to be a distinct form of child maltreatment (Wathen and MacMillan, 2013; Kimber et al., 2018), and it is often included in measures of adverse childhood experiences (Negriff, 2020).

Children’s exposure to IPV increases their risk of internalizing, externalizing, and adjustment problems, with the strength of these associations increasing over time and with broader operationalizations of IPV (i.e., both physical and psychological IPV versus physical IPV alone; Vu et al., 2016). Specifically, compared to their peers, IPV-exposed children and youth are more likely to experience higher levels of depressive and anxiety symptoms; exhibit higher levels of externalizing behaviors, including aggression, bullying, inattention, and impulsivity; and have greater academic and social difficulties (e.g., Fantuzzo et al., 1991; Kitzmann et al., 2003; Osofsky, 2003; Wolfe et al., 2003; Hungerford et al., 2012; Fong et al., 2019; Sharp et al., 2020). In preschool-aged children, IPV exposure is also associated with cognitive deficits, including reduced short-term and working memory capacity and executive functioning (Jouriles et al., 2008a). Further, physiological impacts, including posttraumatic stress symptoms, and more frequent health problems, such as asthma, allergies, and dizziness, have also been observed (Perry, 2001; Kuhlman et al., 2012; Levendosky et al., 2013). Notably, long-term psychosocial impacts have also been demonstrated, with increased relationship challenges in later life and heightened risk of intergenerational transmission of violence, either as victims or perpetrators (Evans et al., 2008; Wathen and MacMillan, 2013; Kimber et al., 2018). In short, it is evident that exposure to IPV interrupts critical developmental trajectories for children and has a multitude of adverse consequences.

Beyond the primary physical and psychological impacts of IPV on survivors’ children, current research on the implications for caregiving behaviors and the parent–child relationship reflects mixed findings. The cascading effects of IPV exposure onto parenting behaviors and subsequently onto child outcomes have been conceptualized using a *spillover* model, which hypothesizes that abusive behavior within parents’ relationships “spills over” into their parenting practices and therefore increases the risk of abusive parenting and disruptive behaviors in their children (Krishnakumar and Buehler, 2000; Whiteside-Mansell et al., 2009). Indeed, the vast majority of researchers have found that parenting quality becomes compromised in households where IPV is experienced, highlighting the evidence linking IPV and negative parenting practices, such as higher levels of harsh discipline, physical abuse, and neglect (Jouriles et al., 2008b; Chiesa et al., 2018), and attachment difficulties (Velotti et al., 2018; McIntosh et al., 2021). Mothers affected by IPV have been observed to experience diminished parental effectiveness and authority and are more likely to demonstrate physical and psychological aggression toward their children. In addition, IPV victimization is linked with negative parenting and maternal psychological functioning (Gustafsson and Cox, 2012).

However, mothers in relationships with IPV have also been shown to make substantial efforts to protect their children and compensate for their children’s exposure to IPV by increasing their sensitivity to their children’s needs (Letourneau et al., 2007; Lapierre, 2010; Chiesa et al., 2018). Indeed, some mothers appear to utilize more effective parenting strategies as well as resilient strategies, such as seeking out additional internal and external resources to promote positive family well-being (Gavidia-payne et al., 2015), suggesting that not all mothers’ parenting becomes compromised (Levendosky et al., 2003). IPV survivors may also paradoxically resort to harsh or physical discipline in an attempt to protect their children from the abuser (Little and Kaufman Kantor, 2002). Put differently, mothers may discipline their children harshly as a protective mechanism to mitigate future violence perpetrated by the abuser by keeping their child “in line.” Most mothers who have survived IPV are fundamentally motivated to be “good” mothers and, against this backdrop, the effects of IPV on their children are often a driving factor in regaining their sense of power as a mother and in seeking help (Lapierre, 2010; Sousa et al., 2021). Qualitative studies have also found that children themselves identify mothers as the most important protective factor in the wake of IPV (Mullender et al., 2002).

Taken together, and considering these protective efforts, sensitive maternal caregiving is an important buffering mechanism against the adverse effects of IPV (Anderson and Van Ee, 2018; Austin et al., 2019). Thus, consistent with resiliency frameworks, several intervention programs have been developed to prevent impairment from IPV exposure by targeting maternal sensitivity and caregiving behaviors. Yet, as we will discuss below, despite the disproportionate rates of IPV exposure among African American women and their children and the rise in IPV associated with the COVID-19 pandemic, an IPV parenting intervention adapted specifically for African American mothers has not been developed.

### Current family-based interventions for IPV survivors

Recent systematic reviews of parenting interventions for mothers and children exposed to IPV (e.g., Anderson and Van Ee, 2018; Lindstrom Johnson et al., 2018; Austin et al., 2019) have identified several programs, including but not limited to Moms’ Empowerment Program (Graham-Bermann and Miller, 2013), Project Support (Jouriles et al., 2001, 2009), and Child–Parent Psychotherapy (Lieberman et al., 2005). These established programs have demonstrated improvements in mother–child interactions and other treatment targets, including significant increases in positive parenting practices (Howell et al., 2015; Grogan-Kaylor et al., 2019), greater reductions in inconsistent and harsh parenting behaviors, and greater reductions in conduct problems (Jouriles et al., 2001; McDonald et al., 2006). Additional positive outcomes of current IPV parenting programs have included reduction in maternal distress and trauma symptoms (Lieberman et al., 2005, 2006). Overall, Lindstrom Johnson et al.’ (2018) meta-analysis demonstrated moderate to large effect sizes for increases in positive parenting practices (*d* = 0.72) and reduction in child internalizing, externalizing, and trauma symptoms (*d* = 0.48–0.59). While additional programs have been developed for mothers and children exposed to IPV (e.g., Kamal et al., 2017; Katz et al., 2020), here our aim is to distill and summarize common characteristics of parenting interventions developed for IPV-exposed families, irrespective of race or ethnicity, to facilitate later integration with the literature on parenting interventions designed for African American women.

First, family-focused IPV programs all necessitate the engagement of caregivers as the primary target of the intervention, with greater variability in the inclusion of children and the extent to which they are involved. IPV programs tend to collect outcome data from children to assess intervention effects but whether children are also recruited as additional subjects to engage in the intervention varies across programs. Further, when both parents and their children are involved as treatment targets, programs use a variety of session formats; namely, separate (i.e., concurrent mother and child groups or counseling sessions), joint (i.e., mothers and children attend the same group or session), or combined (i.e., both separate and joint groups or sessions) sessions. Nonetheless, while the inclusion of children as treatment subjects varies by program and may inform programmatic decisions about session format and content (detailed below), it is clear that an integral component of any family-based IPV program, regardless of race or ethnicity, is the primary engagement of parents in the intervention.

Second, as summarized in Austin and colleagues’ systematic review (Austin et al., 2019), what is common to most IPV-family based programs with respect to intervention themes is the following: improving mother–child interactions and relationship quality, enhancing parenting knowledge and skills, developing adaptive coping and problem-solving strategies, and providing social support to mothers. Treatment targets for mothers tend to focus on improving mothers’ parenting and disciplinary practices, parenting confidence, psychological adjustment and processing of IPV, emotion regulation and coping strategies, and child management skills. Child outcomes include children’s psychological adjustment, behavioral outcomes, social competence, emotion regulation and coping strategies, and understanding of IPV. Programs explicitly designed to target both parent and child outcomes through conjoint parent–child intervention aim to improve the quality of parent–child interactions and/or a combination of the aforementioned parent-and child-specific variables. Overall, the study aims of most IPV programs target behaviors and outcomes in mothers or in both mothers and their children, with less targeting children only (Austin et al., 2019).

Third, family-based IPV intervention programs are often guided by some theoretical orientation that then informs the specific intervention components, which varies by the specific therapeutic framework (e.g., trauma-focused cognitive behavioral therapy, trauma-informed attachment-based, emotion-focused, filial therapy, parent–child interaction therapy, child–parent psychotherapy). For instance, trauma-focused interventions may incorporate some aspect of trauma processing, whereas programs guided by parent–child interaction therapy provide a basis for teaching child management skills while enhancing the attachment relationship.

Another common component of these family-based interventions are the considerations made for the mode of delivery (e.g., in-home, community-based, group format, phone consultation, integration into existing services) and type of intervention facilitators (e.g., social workers, clinicians, mentor mothers), regardless of families’ races and ethnicities. Next, IPV parenting programs often include some element of safety planning, building conflict-resolution skills, and processing IPV exposure, which is common among interventions for IPV-exposed families but a unique feature that differentiates these programs from other psychosocial family interventions developed for broader contexts with non-traumatized populations.

Finally, family-based IPV programs share common recruitment methods, such that mothers tend to be recruited from IPV shelters, the broader community, by referrals from local agencies, or by court mandate. Notably, conclusions from a review by Anderson and van Ee (2018) suggested that the most successful psychosocial recovery interventions for mothers and their children following IPV likely incorporate both separate and joint mother–child sessions and focus on improving mother–child interactions.

Importantly, while research supports the effectiveness of existing parenting programs for IPV-exposed mothers and children, the extent to which these positive effects generalize to African American women and their children is unclear. Specifically, parenting interventions for mothers and children exposed to IPV have been implemented in samples that included African American mothers and children, but analyses of intervention effects for subgroups of families, such as African American families, have not been conducted. As a result, it is unclear if the findings generalize to specific groups. Furthermore, there are reasons to believe that the positive effects of interventions designed for mothers and children exposed to IPV can be improved for African American families. That is, the interventions that have been evaluated thus far do not explicitly incorporate cultural components of African American parenting practices. Family-based programs that attend to the unique sociocultural contexts of diverse families can improve positive parenting and psychosocial outcomes in parents and their children (Coard et al., 2007; Smith et al., 2022). Next, we turn to the literature on African American parenting practices and a discussion of how existing parenting programs for IPV-exposed mothers and children might be enhanced for African American mothers and children.

### African American parenting practices and parenting programs

#### Parenting practices in African American families

African American parents encounter a distinct set of parenting challenges in the United States. While navigating discrimination, racism, limited access to healthcare, disproportionate rates of poverty, unemployment, and incarceration, they must also contend with raising their youth in this environment (Coard et al., 2007). Parenting practices in African American communities differ from other communities in that they consider a range of factors (social, economic, individual) and are aimed to prepare children to navigate societal realities, where they will face and must cope with racial discrimination and systemic inequalities (Kotchick and Forehand, 2002). For instance, central to African American parenting is the use of racial socialization practices; the socialization process by which parents transmit verbal and non-verbal messages about the significance of their racial/ethnic group, and their children subsequently “acquire the behaviors, perceptions, values, and attitudes of [their] ethnic group, and come to see themselves and others as members of the group” (Phinney and Rotherham, 1987, p. 11). For African American families, racial socialization practices are those that teach youth what it means to be Black by instilling a strong sense of African American culture and pride, explaining the dynamics of inter-and intragroup interactions, and preparing them for the negative race-related challenges they will face (Stevenson, 1994; Lesane-Brown, 2006). Growing evidence suggests that African American youth experience better psychosocial outcomes when their parents use more racial socialization practices (Coard et al., 2004; Wang et al., 2020). In addition to these socialization practices, shared parenting responsibility with extended family members, and having spiritual or religious faith are highly valued within the African American community (Coard et al., 2004). These constitute important supports for African American youth, as they promote healthy psychological well-being and positive self-concept, reduce risk-taking behaviors, and serve as a coping mechanism against racism and discrimination (Butler-Barnes et al., 2018; Rose et al., 2020).

Prior literature has demonstrated that African American parents tend to use physical disciplinary strategies more frequently than White parents and are more likely to be described as authoritarian, a parenting style characterized by low warmth and high control through restrictive parenting practices that utilize harsh, punitive disciplinary methods (Baumrind, 1971; Maccoby and Martin, 1983). In light of this, studies on African American childrearing strategies often evaluate the impact of authoritarian parenting practices and other indices of parental control, including strictness, monitoring, intrusiveness, physical discipline (e.g., spanking), and demanding behaviors on African American children (Tamis-LeMonda et al., 2008). While an authoritarian parenting style tends to be associated with negative child outcomes in White youth, outcomes have resulted in mixed findings for African American children. For instance, higher levels of parental physical discipline have been found to predict higher levels of externalizing behaviors among White children but lower levels of aggression and externalizing behaviors in African American youth (Deater-Deckard et al., 1996). Conversely, other findings indicate that the authoritarian style and its characteristics are associated with negative outcomes for both White and African American children (Kelch-Oliver and Smith, 2015). It is also noteworthy that African Americans’ use of physical discipline stems from the institution of slavery, wherein parents used harsh discipline to promote their children’s survival (Bradley, 1998; Thomas and Dettlaff, 2011; Kelch-Oliver and Smith, 2015), and is continued even now, given the continued presence of racism, white supremacy, antiblack rhetoric, and police killings of Black people (Patton et al., 2021).

Despite mixed evidence of authoritarian parenting and harsh discipline on African American children, research with African American youth has pointed to the universality of the positive benefits of supportive, responsive parenting behaviors (e.g., Elmore and Gaylord-Harden, 2013). As a result, parenting interventions aimed at increasing parental sensitivity, responsiveness, and warmth and reducing harsh, controlling disciplinary practices to promote positive child outcomes have been developed for African American parents-aims that are consistent across several programs for families in which there has been IPV. Further, a common theme in these tailored parenting interventions is the promotion of racial socialization practices and recognition of the “broader historical context and functional significance of physical discipline in African American culture” (Kelch-Oliver and Smith, 2015, p. 27). We highlight key culturally-relevant components of select African American parenting programs, which are distinct from many “standard” parenting programs for IPV-affected families, in the forthcoming section. Of note, these select parenting programs were included based on our literature review of parenting interventions for Black and African women, which yielded studies based on a search strategy using a combination of search terms and their adjectives or derivatives (*African American, Black, mothers, families, parenting intervention, parent training*) and review of reference lists of relevant articles. The parenting programs described below include those identified in our review that provided details of the intervention components, evaluated treatment outcomes, and/or described the process of cultural adaptation.

#### African American-focused parent-training programs

The Effective Black Parenting Program (EBPP; Alvy, 1987; Alvy and Marigna, 1990) is a cognitive-behavioral, skills-based program designed specifically for parents of African American school-aged children to improve parental behaviors (e.g., reducing coercive disciplinary practices, increasing positive parenting practices), parent–child relationship quality, and child behavioral and socioemotional outcomes. Parents are encouraged to explore the functional purpose of their rules and provide this rationale to their children (i.e., “appeal to their minds and not their behinds”) and to consider the developmental and environmental causes of their children’s misbehaviors (Myers et al., 1992). Additionally, parents are taught specific behavioral management strategies for increasing desirable behaviors, such as using behavior-specific praise and small incentives, and reducing undesirable behaviors, such as time outs and ignoring. Importantly, the EBPP carefully considers and integrates several aspects of African American culture into its framework and delivery. In line with racial socialization practices, parents are encouraged to instill *pride in Blackness* (Myers et al., 1992) in their children while also helping them to cope with race-and ethnicity-related issues. Discussions of coercive parenting practices (i.e., physical discipline) are sensitive to the historical significance and adaptive utility of these practices and acknowledge their contemporary use as protective measures against dangerous environments and the race-related consequences of violating social norms. However, through meaningful discussions parents are motivated to engage in positive parenting behaviors and challenged to view these traditional disciplinary strategies as potentially interfering with their children’s ability to become empowered individuals who can effect social and economic change (Myers et al., 1992). Finally, the EBPP is culturally-sensitive in its program delivery in that it is exclusively delivered by African American professionals. Regarding treatment outcomes, Myers et al. (1992) reported mixed findings on two cohorts of families participating in EBPP. Treatment parents reported significant pre-posttest reductions in parental hostile/aggressive rejection and parental undifferentiated rejection compared to control parents in both cohorts, with an observed trend for reductions in these specific parenting practices at 1-year follow-up, though not statistically significant (*p* < 0.07). There was also mixed evidence of significant group differences in improvements in family relations or child behavior outcomes in the two cohorts, as well as a surprising finding of regression to aggressive parenting practices among treatment parents at 1-year follow-up. Thus, while the EBPP is among one of the first culturally adapted interventions for African American families, that its evidence base has few sustained statistically significant findings is a notable program limitation.

The Black Parenting Strengths and Strategies (BPSS) Program (Coard, 2003; Coard et al., 2007) is another culturally-relevant, strengths-based parenting program that was adapted from the Parenting the Strong-Willed Child Program (Forehand and Long, 1996) and designed to support African American caregivers. Leveraging the unique parenting strategies of the African American community (e.g., parental racial socialization) and the specific social, cultural, and political challenges that impact parenting in this community, the BPSS program focuses on strengthening parenting skills, involvement, and confidence to reduce children’s behavioral and socioemotional problems (e.g., noncompliance, conduct disorders). Specifically, parents are encouraged to engage in positive parenting, racial socialization, and parental monitoring practices to promote positive self-image in their children, positive discussions with their children about race, increased academic confidence in their children, and enhanced parental understanding of their children’s socioemotional development (Coard et al., 2007). In addition, parents are allotted a significant portion of each group session to discuss their experiences as African American individuals and as parents with one another. Cultural considerations are also made for program delivery and possible barriers to participation. Groups are led by African American women and both childcare and meals are provided during the sessions. Finally, there are several modifications to the program materials, including the use of common African American language and expressions, *we-*talk to represent collectivist African American values, sayings and affirmations that are meaningful to this community, prayer, role-playing, storytelling, including extended family members, and humor (Coard et al., 2007). The BPSS program has been shown to be successful in significantly increasing parental use of racial socialization and positive parenting and reducing harsh disciplinary practices and child externalizing problems (Coard et al., 2007). Further, the average attendance rate across 12 sessions was 85%, 88% of all families (intervention and waitlist-control) completed the post-intervention assessment, and 100% of intervention families completed the post-intervention assessment. These attendance and retention rates are remarkable given the difficulties in recruiting and retaining African Americans for family-based programs (Kumpfer et al., 2002). Limitations of the BPSS program include the use of a small sample size (*N =* 30) and provision of participant compensation, limiting the generalizability of findings.

The Strong African American Families (SAAF) Program is a 7-week preventive program developed for rural African American mothers and their 11-year-old children (Brody et al., 2004). Through the promotion of effective parenting behaviors and communication strategies (i.e., racial socialization, involved-vigilant parenting, communication about sex, and articulated expectations for alcohol use), this intervention aims to enhance youth self-pride and deter them from risky behaviors—specifically, alcohol use and early sexual debut. The weekly group sessions, which consist of separate parent and youth groups and a joint group at the end of each session, are led by African American facilitators and held in local community centers. To address potential barriers to treatment, transportation, childcare for siblings, and a catered meal are provided. Evaluations of SAAF confirm that, compared to the control group, intervention youth were less involved in alcohol use, sexual risk-taking behavior, and conduct problems across time, and parents exhibited increases in communicative parenting strategies (Brody et al., 2004, 2008; Murry et al., 2007). However, that the sample was limited to 11-year-old children in rural areas is one limitation of the SAAF program, as these findings may not be generalizable to African American children from different age groups and regions with different community characteristics (e.g., neighborhood composition, inner city versus rural community settings) (Tamis-LeMonda et al., 2008).

Gross et al. (2009) developed the Chicago Parent Program (CPP), an evidence-based, culturally-tailored intervention for parents of preschool-aged children, in consultation with an advisory board of low-income African American and Latinx parents. This 12-week group intervention is intended to increase positive parenting skills, improve parental competence, reduce harsh disciplinary practices, and reduce preschoolers’ behavioral problems through the use of videotaped vignettes of real-world parenting scenarios (e.g., parent–child interactions at home, in the grocery store) to guide group discussions about specific parenting behaviors (Gross et al., 2009). Importantly, feedback from the advisory board was integral to making decisions regarding how to present and discuss specific components of the intervention in a culturally sensitive, relevant, and meaningful way (e.g., offering alternative disciplinary strategies to physical punishment rather than taking a hard stance against it). The program also allows group facilitators to flexibly discuss core CPP concepts in culturally-specific ways, such as discussing stress management in the context of also having to cope with the stress of racism. In terms of logistics, CPP has been implemented in both professional and community settings (e.g., schools, local community centers) and offers program participants meals or refreshments and childcare for siblings. Participation in CPP resulted in less parental use of corporal punishment and child commands and fewer observed child behavior problems, as compared to participation in the control condition (Gross et al., 2009).

Similarly, another group parenting program for parents of African American adolescents in urban, high-crime neighborhoods was adapted to this target population through a variety of techniques (Finigan-Carr et al., 2014). The techniques included input from a parental community advisory board comprised of African American parents, the use of *photo novella* story essays to depict specific concepts using a sequence of images and storyboards of interactions between African American parents and their youth, acknowledgment and discussion of parenting stress in urban environments, accommodation of families’ schedules by offering sessions at various times and in various community locations close to participants, and offering transportation vouchers, meals, and childcare. Results from a feasibility study revealed that only 34% of parents who consented to participate were also enrolled in the intervention, and intervention participation was highest among parents receiving in-home session visits compared to parents receiving community-based group sessions or parents participating in a hybrid model (Finigan-Carr et al., 2014).

Other interventions have taken established parent training programs that were not initially developed for or tailored to African American parenting contexts and have implemented them in exclusively African American communities or diverse populations that include African American parents. For instance, the Triple P-Positive Parenting Program (Triple P; Sanders, 1999) was evaluated in a case study of a low-income, African American mother of a preschool-aged child (Kelch-Oliver and Smith, 2015). In this study, a set of recommendations for adapting Triple P to low-income, single African American parents was put forth, including ensuring audiovisual materials are representative of the target population, modifying wording in the manual to be more culturally sensitive, being cognizant of parents’ literacy and education levels, being sensitive to the race-related factors associated with harsh discipline, using group facilitators who are culturally competent and/or of the same culture and can interject anecdotal experiences, including other important parental figures, and offering telephone consultations and post-intervention booster sessions. Child parent relationship training (CPRT; Bratton and Landreth, 2006) is a strengths-based program that utilizes play to facilitate parental understanding of their children and has also been implemented with low-income Black and African American parents (Sheely-Moore and Bratton, 2010). Finally, an in-home adaptation of a parent and child therapy (PCT) program has been conducted with a diverse sample of African American, Latino, and White parents of young children with significant behavior problems to address barriers that low-income, culturally diverse groups often face (e.g., lack of transportation and childcare) by making the intervention transportable to a naturalistic environment (Gresl et al., 2014). Clinicians teach parents alternative ways of responding to their children in an effort to reduce childhood behavior problems and harsh parenting techniques. In addition, clinicians use an appropriate and matched communication style with the parents.

Researchers have also conducted qualitative interviews and focus groups with African American parents to understand their parenting views and solicit their feedback about the needs of their communities and the specific considerations to be made when developing culturally-relevant parenting programs. Several themes related to parental racial socialization emerged from qualitative interviews conducted by Coard et al. (2004). Specifically, the content of mothers’ race-related messages conveyed meaning related to racism preparation, racial pride, racial equality, and/or racial achievement, and was communicated via oral communication, modeling, role-playing, and/or exposure (Coard et al., 2004). Unequivocally, racial socialization practices were considered to be a critical component of African American parenting. Focus groups with African American and Latino parents have revealed various perspectives on parenting-related challenges (e.g., cultural differences in parenting, childcare laws and policies), the need for parenting education in their communities (e.g., informing parents of acceptable parenting practices in the United States, providing a space for parents to network and share their experiences), and the shortcomings of existing parenting education programs (e.g., no cultural components) (Toure et al., 2021). Recommendations for the ideal parenting program include ensuring the program is culturally appropriate, providing updated parenting information and education on child development and legal issues related to childrearing strategies, program delivery by community members familiar with the culture, recognition of different family structures and circumstances, discussion of discipline, religion, education, and community resources, and assurance to discuss sensitive topics without fear of being disciplined (Toure et al., 2021).

Finally, it is noteworthy that among the parenting programs that have been specifically tailored and adapted for and/or evaluated in African American communities, the primary goal of these interventions has been to enhance parenting behaviors, reduce coercive disciplinary practices, and reduce or prevent children’s socioemotional or behavioral challenges. These programs commonly integrate the following elements: a strengths-based approach, racial socialization practices, discussion of race-related challenges, consideration of the historical context of physical punishment, group sessions led by African American facilitators, held in the community, audiovisual and linguistic modifications to program materials, and provision of meals, transportation, and childcare. What primarily distinguishes these programs from one another lies in the specific child outcome the intervention aims to address (e.g., broad psychosocial and behavioral challenges versus specific risky behaviors). Further, the extent to which these interventions consider the specific parenting contexts that African American parents encounter is substantially limited (e.g., non-resident fathers).

### A roadmap for tailoring IPV parenting interventions for African American mothers

Head-to-head comparisons of culturally adapted parenting interventions to non-adapted parenting interventions are scarce (McCabe et al., 2012). However, results from meta-analytic studies generally suggest that culturally adapted mental health interventions improve program relevance, acceptability, and sustainability and are more efficacious for ethnic minorities-though with variability in effect size estimates-when compared to non-adapted intervention, no treatment, and treatment-as-usual conditions (Griner and Smith, 2006; Benish et al., 2011; Cabassa and Baumann, 2013; Baumann et al., 2015). In the absence of studies that have explicitly adapted IPV parenting programs for African American women, the current paper aims to bridge this gap by presenting a set of guiding principles, recommendations, and reflective questions for future IPV parenting interventions to be culturally adapted to the African American context. In so doing, this deliberate synthesis of African American parenting practices and values with core IPV family-based intervention components will allow IPV parenting researchers to make use of this framework and adapt existing interventions for this priority population in a manner that is intentional and culturally sensitive.

Critically, we posit that any parenting intervention for African American IPV-surviving mothers would benefit from explicit consideration of the unique parenting challenges mothers grapple with, such as incorporation of racial socialization practices, and that interventions must sufficiently acknowledge and be tailored to allow for discussions and engagement with these unique challenges. To assist this intervention development and adaptation process, we propose a framework that highlights seven key principles and is informed by our review of the literature (see Table 1). To guide our development of these seven guiding principles, we largely employed a data-driven, bottom-up approach using the themes we identified as common among parenting interventions for African American parents. However, we also allowed our development to be guided by intersectionality and social justice frameworks. Importantly, while we consider these suggestions to be of equal importance, we recognize that incorporating all suggestions may not be feasible and encourage programs to evaluate which suggestions are within the scope of their project. Given this, we also put forth a series of questions for consideration as they pertain to each principle. Supplementary Table 1 lists these illustrative questions.

**Table 1:** Seven guiding principles for adapting IPV parent–child interventions for African American mothers.

1. Using a strengths-based approach to avoid perpetuation of discrimination against African American women as caregivers
2. Integrating racial socialization specific to African American interests and values
3. Embracing African American cultural values and traditions
4. Addressing barriers through equity promotion focused on disparities African American women face
5. Ensuring a safe space and transparency about mandated reporting sensitive to African American disciplinary practices
6. Adopting peer support networks for African American survivors of IPV
7. Providing psychoeducation and resource sharing to address specific gaps identified for African American mothers and children

#### Using a strengths-based approach

Historically, the quality and nature of African American families have been viewed from a deficit lens. From this theoretical perspective, African American families’ experiences are conceptualized as the result of innate deficiencies that perpetuate high poverty, unemployment, crime rates, single-parent families, and welfare recipiency, and the traditional matriarchal structure is thought to be the mechanism by which families get “caught in a tangle of pathology” (Briscoe, 2000, p. 98; Smith et al., 2022). This perception of African American parents carries into school systems, where they are perceived as uninvolved, unconcerned, and threatening by educators for not demonstrating investment in their children’s education according to traditional conceptualizations of parental involvement (e.g., physical presence at school events, homework assistance, communication with teachers, monitoring academic performance) (Love et al., 2021). Consequently, interventions aiming to promote academic, behavioral, and socioemotional outcomes in African American children and improve parenting strategies among African American parents have predominantly been guided by a deficit-based approach. As a result, the reality of historical trauma, systemic and institutional racism, discrimination, and the inherent strengths of African American parents are largely dismissed, minimized, or ignored. Moreover, an implicit message of helplessness and the need for external resources to drive the solutions to their challenges is conveyed.

Despite the extensive literature base using a deficit model to understand African American parenting, current perspectives on parenting have shifted away from this framework and have begun to move toward strengths-based, or asset-based, approaches that embrace individuals’ unique abilities and focus on positive, protective, and resilient factors, as opposed to individual shortcomings and difficulties (Briscoe, 2000; Pollock et al., 2015). For instance, the authoritarian style attributed to African American parenting is now better understood as an example of the heterogeneity in parenting within the African American community which is influenced by the harsh sociopolitical realities they face, rather than a deficient parenting style that is characteristic of the African American community. The family strengths perspective more reasonably works toward understanding how “families succeed in the face of life’s inherent difficulties” and further conceptualizes the difficulties families encounter as “vehicles for testing our capacity as families” (Asay et al., 2016). Such approaches are strongly recommended by researchers, as they tend to be multifaceted and acknowledge the social and political systems that impact parenting abilities (Sousa et al., 2021). We argue that parenting interventions explicitly utilizing a strengths-based lens are especially well-poised and conducive to working with IPV-exposed population – specifically, African American women-as a way to ensure the challenges that these mothers face as both African American women and as IPV survivors, such as stigmatization and inadequate welfare programs, and the ways in which they succeed as parents are not overlooked, minimized, or dismissed. Such a framework is sensitive to these intersecting identities and should make use of African American mothers’ existing parenting abilities and the cultural values most salient to them by emphasizing that there is significance and power in their core beliefs and values about parenting, family, and culture, regardless of their trauma exposure. By bringing the inherent strengths and values of African American mothers to the forefront, strengths-based programs can effectively enhance their feelings of competence and motivate their engagement with the intervention. We encourage programs to consider how they can incorporate messages of parental strength, resilience, and competence into the interventions’ rhetoric and within the relationships that intervention facilitators establish with participants and that group members establish with one another, while maintaining the integrity of a strengths-based versus deficit-based approach. Moreover, we contend that this reflection should be frequent and ongoing throughout the intervention delivery period to ensure the program remains grounded in the strengths-based framework. To guide this reflection, the following questions are set forth.

1. Are intervention facilitators engaging with mothers and asking what parenting values are most salient to them and their community, or have these values already been assumed?
2. Are intervention facilitators inviting mothers to identify the ways in which they are succeeding as parents in their own, unique contexts and then using this as an opportunity to enhance their sense of competence, or is a model of parenting being imposed?
3. Is an appreciation for the mothers’ opinions and experiences being conveyed via frequent check-ins throughout the intervention and at its conclusion about what worked and did not work, or are their voices being dismissed?

#### Integrating racial socialization

As outlined in our examination of African American parenting programs, parenting practices within the African American community are unique and distinct from those employed in non-African American communities in their use of racial socialization strategies. African American parents are tasked with not only navigating their own daily experiences of racial discrimination and trauma but also equipping their children with the psychological armor to cope with and navigate the same experiences they will come to encounter (Lesane-Brown, 2006). Within various frameworks for racial socialization, several themes have been identified related to the types of experiences and messages conveyed, rationale for these practices, and modes of teaching (Hughes et al., 2006). For African American children, they commonly receive both implicit and explicit messages about cultural heritage and pride, personal and group identity, inter-and intra-group relations, African American experiences versus “mainstream” European American experiences, social hierarchy, and education about the historical and modern-day context of racism (Coard et al., 2004; Lesane-Brown, 2006). Thus, parenting interventions for IPV exposed mothers and children would potentially benefit from the incorporation of racial socialization practices.

The Black Parenting Strengths and Strategies Program (BPSS; Coard et al., 2007) is one exemplar intervention that tapped into the importance of racial socialization strategies for African American parents by incorporating “culturally specific content and delivery strategies concerning racial socialization” (Coard et al., 2007, p. 805) without diluting content from the original parenting intervention. Specifically, the BPSS program allotted time during the parenting group sessions to discuss specific race-related challenges, process parents’ individual and group experiences as African Americans, and learn about developmental processes for racial identity formation (Coard et al., 2007). Importantly, these adaptations were informed by a qualitative analysis identifying the common race-related messages parents give their parents have also been identified, which included parents’ efforts to instill racial pride, prepare their children for future encounters with racism, impart multi-ethnic egalitarian values, and emphasize the need to excel and achieve (Coard et al., 2004).

Against this background, we provide the following reflection questions to assist researchers in their considerations of how to incorporate racial socialization strategies into their interventions.

1. Does the intervention make space for parents and children to talk about their racial identities and personal experiences? Do facilitators touch on aspects regarding racial pride, preparation for racial prejudice, achievement, and racial equality?
2. Does the intervention explore what it means to be Black in America, especially within the unique contexts of the participating families (IPV exposure), and how does it acknowledge the historical and modern-day implications of racism?
3. To what extent are these conversations being incorporated into the intervention content? Are these stand-alone modules to be discussed in addition to the “standard” parenting curriculum, or have the two “contents” been fused together? For group discussions, what will this look like (e.g., built-in discussion time during each session, in session intervals, as needed)?
4. What are the mechanisms for transmitting racial socialization messages the intervention is using? Are both explicit and implicit methods employed?

#### Embracing African American cultural values and traditions

In order to ensure that interventions truly meet the needs of their target population, they must be sufficiently designed or adapted in a manner that includes both ‘surface structure’ changes to intervention materials and messages that match the observable characteristics of the target population and ‘deep structure’ adaptations, which concern “incorporating the cultural, social, historical, environmental, and psychological forces that influence the target health behavior in the proposed target population” (Resnicow et al., 2000, p. 271). In doing so, target populations may be more receptive to the intervention program and perceive it as being more salient to their personal experiences and their community (e.g., Coard et al., 2007). For the purpose of intervention development and adaptation for IPV-exposed African American families, in addition to integrating racial socialization practices, researchers would benefit from incorporating other components that demonstrate an appreciation for the cultural values and traditions of their community while also ensuring the more “visible” adaptations are in place. These surface changes include adapting audiovisual material to make sure images are culturally representative and words are linguistically appropriate and that intervention facilitators are of similar backgrounds, or at the least, knowledgeable and caring toward this population.

Regarding the deep structure adaptations, apart from racial-ethnic socialization practices, it is well-established that strong spirituality and religion plus a reliance on extended family networks and *fictive kin* are culturally salient resources for African American families (McNeil Smith and Landor, 2018). Accordingly, family-based interventions for African American parents can embrace these core values by recognizing these as strengths and making space for them in their intervention programs, which can include having extended family members as participants or incorporating spiritual or religious messages into program rhetoric. With respect to trauma-exposed families, integration of these values and traditions may be especially important to instilling messages of strength and resilience and demonstrating program relevance for their community, as it aligns with what we know about African American mothers’ help-seeking experiences following IPV (Waller et al., 2022). That is, we know that African American women often lean on their churches for guidance and support rather than mental health providers or criminal justice sectors, and that extended family members often play an important caregiving role in their children’s lives, especially in the aftermath of IPV exposure as they navigate the structural barriers that evoke re-traumatization. Finally, of note, the voices of program participants, past and present, and other members of the community (e.g., community advisory boards) can also be particularly helpful in guiding researchers to make these decisions regarding the values most salient to this community and how to adapt program elements to be the most well-received by future program participants. Given these considerations, we propose that researchers consider the following questions.

1. Who is included in the caregiving support system of the parents participating in the intervention (e.g., grandparents, godparents, aunts), and are these members included in discussions?
2. Are there strong spiritual, religious beliefs that guide the parenting practices of the participants? How does the intervention ensure that it is respectful of this, and to what extent are parents encouraged to harness these strongly held beliefs to enhance their parenting?
3. Are program materials sufficiently and appropriately adapted specifically for African American mothers and children? How are materials evaluated to be culturally relevant and sensitive? Has feedback been sought from members of the community?
4. Who is facilitating the intervention – a member of the community with shared experience or an outside member in a position of power despite having little or no knowledge of the community?

#### Addressing barriers through equity-promotion practices

Equity is defined as the “absence of avoidable, unfair, or remediable differences” between groups of people (World Health Organization, n.d.). To promote equity is to reduce disparities between groups, including the removal of barriers in accessing resources and interventions. Thus, equity-promotion practices are an essential component of interventions intended to benefit vulnerable populations, and care must be taken by intervention developers and providers to ensure equitable access to their interventions, regardless of the economic, practical, or other challenges their target populations encounter. For interventions targeting IPV-exposed African American mothers, it is critical that equity-promotion practices be incorporated to mitigate the multitude of barriers they face, which not only impact mothers’ accessibility to interventions but also their level of engagement and participation. In other words, parenting interventions for these mothers must aim to address both the structural (e.g., lack of transportation, childcare, and time) and attitudinal (e.g., lack of parental buy-in regarding program relevance) barriers (Kerkorian et al., 2006). Without equity-promotion practices, these barriers may preclude parents from participating in the intervention altogether, which influences the number of persons reached by the intervention and the ability to assess whether the intervention is effective for all members of a targeted group. For example, if an intervention requires in-person attendance at a group meeting but does not provide transportation, it is likely that the number of individuals who cannot afford cars, bus fare, or rideshare may be excluded from the intervention. This exclusion not only grossly highlights and perpetuates the systemic inequalities these vulnerable families already face, but it may also impact the generalizability of the research findings, as there may be systematic differences in families who do have access to transportation and those who do not. On the other hand, providing transportation for those who need it, whether through monetary assistance or pick-up services, is an equity promotion practice that increases the external validity of the intervention. Put differently, the removal of barriers (in this case, transportation), increases our ability to deliver interventions to targeted groups and our ability to assess the impact of interventions. Moreover, it taps into the community-building aspects of equity promotion by bringing in more families from the same community to participate in the intervention. The provision of childcare, meals, and technological resources as well as intervention delivery by paraprofessionals in non-traditional settings are among other common implementation strategies used in community-based interventions for hard-to-reach families that harness the community-building benefits of equitable interventions (Cooper et al., 2022). Ultimately, it is in programs’ best interest to make it as easy as possible for already busy and vulnerable families to participate in their intervention.

We put forth the following guiding questions for intervention developers to consider in order to promote equity in their intervention development, as well as maximize validity of their study such that potential participants are not systematically excluded as a result of their barriers faced.

1. What factors might prevent a potential participant from participating in the intervention? For instance, is the program time/location convenient to them? Do participants require childcare? Is it expensive for participants to attend? Will participation in the intervention interfere with participants’ work or other obligations (e.g., cooking a meal)?
2. Given relevant barriers, how can the intervention be modified or adapted to promote equity for participants? Can transportation be provided? Is it possible to convert some in-person requirements to virtual? Can relevant technology be provided if the intervention necessitates it? Can childcare or meals be provided? Can parking, gas, or bus fare be reimbursed?
3. How can intervention fidelity and adherence be maintained while also promoting equity? Note that this ratio will be different for every intervention and population and will also depend on the goals of the intervention developer. In order to address every barrier for a population, it is possible that the scope will be narrowed due to costs. If it is needed to cover transportation, technology, childcare, and meals for participants, there may be fewer participants reached in total.

#### Ensuring a safe space and transparency about mandated reporting

Given the focus of these recommendations on African American families impacted by IPV, the discussion and treatment of child abuse allegation is critical. Depending on state and local laws, many intervention developers and providers may be required to report any suspicion of physical abuse of a child, including physical punishment. Mandated reporting can present a challenge for providers and organizations, given its potential custody and legal impacts, in the context of relationships with clients. Indeed, research has shown that many mandated reporters have low levels of reporting to Child Protective Services (CPS; Alvarez et al., 2004). Further, many experts even disagree about the standard threshold for reporting (Levi and Crowell, 2011). These challenges with mandated reporting are relevant for all providers working with children and families, but they are especially relevant in working with African American families who have been exposed to IPV. Research with African American families has demonstrated increased rates of authoritarian parenting practices, including physical punishment (Taillieu et al., 2014). As mentioned previously, several factors influence this parenting practice, including the historical context of violence toward African Americans in the United States (Thomas and Dettlaff, 2011) and the modern-day context, which includes state-sanctioned discrimination and violence, including by the criminal justice system (Durrant, 2008). Against this background of increased use of physical punishment, it should also be noted that African American women are also at risk for retraumatization and suffer worse legal and custody outcomes than other groups.

Mandated reporting and concerns related to physical abuse and safety are crucial issues for parenting interventions to address. We suggest the following questions to guide intervention developers and providers to maximize transparency with the clients they serve while allowing for honest dialogue, as well as maintaining the legal and ethical responsibilities associated with keeping children safe.

1. What are the local/state/federal guidelines for mandated reporting? What constitutes physical abuse? Who needs to be informed when a report is made?
2. How can I ensure my clients are aware of the requirements I must abide by? At the start of each session, can I explicitly remind them of what is and is not reportable?
3. If a CPS report must be made, how can the parent be informed of this process in a manner that is sensitive and works to maintain the relationship between the parent and the intervention provider? How can the shared priority of the child’s safety be leveraged in the provider-parent relationship?

#### Adopting peer support networks

Our review of interventions for families affected by IPV as well as family-based interventions for African Americans points to the importance of the dual roles parenting interventions have in not only giving parents a forum to acquire knowledge and parenting skills to improve their own and their children’s psychological and behavioral health outcomes, but also provide a space for mothers to connect with one another and process their experiences. This opportunity for African American mothers to seek support from members of their community with shared experiences and to be a reciprocal source of support is a critical component of these interventions (Toure et al., 2021). Indeed, *sister circles* are a common style of support group in the African American community (Neal-Barnett et al., 2011). Consequently, it is essential that IPV parenting interventions adapted for African American families build on this relational foundation and aim to cultivate lasting relationships that extend beyond the immediate intervention group context. This can be done by adopting a relational orientation that recognizes the importance of relationships both within intervention groups (i.e., relationships with one another and with intervention facilitators) and between intervention groups (i.e., relationships formed by connecting participants with former participants and intervention facilitators, akin to mentoring or coaching one another). Put differently, adopting a peer support network creates both cohort-level relationships and a broader sense of community among former and current group members that contributes to the support system that parents need for their own psychological well-being and to enhance their parental efficacy. To this end, researchers may consider reflecting on the following questions.

1. Does the intervention take a relational lens that aims to build foundational relationships between group members, both former and current, and intervention facilitators? Has the intervention been intentional in bringing together members of the community with shared cultural and racial experiences?
2. To what extent and how does the intervention aim to cultivate relationships? Have we established a model for “graduates” of the program to stay connected with each other and future cohorts? Do we stay in touch with families both formally (e.g., telephone consultations, booster sessions) and informally?
3. If the intervention incorporates a group processing element, what will this look like? Do we intend to integrate sister healing circles? How do we strike a balance between allowing enough time for group conversations to be healing and empowering while also being mindful of intervention adherence and curriculum needing to be covered?

#### Providing psychoeducation and resource sharing

Empowerment is an important component of many IPV-related services. Empowerment has been operationalized in the IPV field as a process in which a person sets goals to gain power and control over their lives by accessing and developing skills, community resources and supports, and self-efficacy (Cattaneo and Chapman, 2010). This process also includes gaining access to knowledge, especially information that is relevant to specific circumstances for African American mothers who have survived IPV. As a trusted service provider, parenting interventions have the opportunity to connect parents with important resources to maximize the growth potential of their children and help families achieve their goals. These resources may include educational information on developmental milestones for parents of young children, adolescent development and relationships for parents of teenagers, and on the psychological and neurocognitive impacts of IPV. Parents may also find it useful for parenting interventions to serve as a resource sharing hub that they can turn to for assistance with finding various services (e.g., employment, food assistance, housing, other social services). More information shared with parents will empower them to make better, more informed decisions for themselves and their families. Therefore, we propose the following critical questions:

1. What questions have families asked that are outside the scope of the intervention content? What information are they seeking or have they conveyed interest in learning about?
2. What services does this target population need that the intervention can help put them in touch with?

## Discussion

In synthesizing the two disparate literature bases on African American parenting interventions and interventions for families surviving IPV, we have provided a ‘roadmap’ for tailoring parent–child interventions for IPV-exposed African American families; also setting forth seven recommendations, each with a corresponding set of reflective questions for researchers to consider. These include: (1) Using a strengths-based approach to avoid perpetuation of discrimination against African American women as caregivers, (2) Integrating racial socialization specific to African American interests and values, (3) Embracing African American cultural values and traditions, (4) Addressing barriers through equity promotion focused on disparities African American women face, (5) Ensuring a safe space and transparency about mandated reporting sensitive to African American disciplinary practices, (6) Adopting peer support networks, and (7) Providing psychoeducation and resource sharing.

Importantly, our proposed framework shares similarities with the common components we identified in our review of IPV family-based interventions regardless of race and ethnicity, while also bearing important differences. For instance, engagement of parents as the primary target of the intervention was identified as a common component, which although not explicitly stated in our framework, is implied by our proposal of Principle 2 (integration of racial socialization), which highlights the promotion of a specific parenting practice. Similarly, a common intervention theme among IPV family-based programs is providing social support to mothers, which is subsumed under Principle 6 (adopting peer support networks) of our proposed framework. Common considerations by most IPV programs regarding the mode of intervention delivery (e.g., in-home, community-based, integration into existing services) are also captured by our guiding principle of addressing barriers through equity promotion (Principle 4). However, under our model, this is reflected upon in greater detail with respect to the disparities that African American women face, so that we can provide practical suggestions. Several IPV programs are also guided by some theoretical orientation (e.g., trauma-focused, attachment theory). Likewise, our proposed framework is informed by theoretical lenses including intersectionality (Crenshaw, 1998) and a social justice perspective (Morris, 2002). Finally, it is important to note that while the common elements of IPV family-based programs and our guiding principles have some overlap, there are considerable differences. Most obviously, our considerations of the unique parenting challenges of African American families due to their overrepresentation in IPV and criminal justice system directly informed each guiding principle within our framework, whereas common components of IPV family programs are generally agnostic of race or ethnicity and consideration of whether they can be made to “fit” the African American context is typically a secondary step. Other key distinctions include the explicit designation of a strengths-based approach, incorporation of African American cultural values and traditions, and sensitivity of the historical roots of physical discipline with respect to mandated reporting.

While each recommendation aims to address some unique aspect of African American IPV-exposed families’ experiences and is therefore considered of equal importance, we note that in practice, the incorporation of all suggestions may not be feasible and is thus a potential limitation of our framework. However, in these circumstances, we encourage intervention developers to use the guiding illustrative questions to facilitate discussions with their teams, considering input from individuals at multiple levels of involvement in the project (e.g., principal investigators, intervention facilitators, community stakeholders, community advisory boards, funding agencies). In particular, soliciting feedback from community advisory boards composed of members from the specific community the program aims to serve, as well as from community stakeholders with prior experience serving this community, can be informative for determining which guiding principles hold the greatest importance. Because an individual program’s ability to make specific cultural adaptations will be unique to their setting in terms of project aims, personnel, location, community involvement, funding resources, and characteristics of their specific community, we recommend first identifying the factors most salient to the program’s context (e.g., specific barriers in their setting, available resources allocated for their program, desired resources for program implementation, previous experience working with and knowledge of the community, needs of the given community) and then reflecting on the feasibility of each component of our framework, given these salient factors. These domains can be conceptualized and assessed using the Consolidated Framework for Implementation Research (Damschroder et al., 2022). Further, as cultural adaptation is a critical aspect of implementation science (Baumann et al., 2015), additional frameworks such as the Ecological Validity Model (Bernal et al., 1995) can be utilized iteratively to enhance sustainability and feasibility. Reflections based on the present guiding principles should remain ongoing and collaborative given that cultural adaptation and implementation are both dynamic processes that may require adjustments throughout the intervention (Cabassa and Baumann, 2013).

Taken together, our goal in setting forth seven guiding principles and reflective questions was to provide a potential solution to the current limitations of IPV parenting programs, which do not address the specific needs and challenges of African American families. We acknowledge our recommendations and reflective questions are process-oriented in lieu of content-based suggestions for addressing specific parenting behaviors. This is done intentionally, as we believe this is consistent with a strengths-based perspective (Principle 1) that draws from the unique characteristics and strengths of individuals from all levels of involvement of an intervention program (e.g., parents/participants, facilitators, supervisors) as opposed to an orientation that imposes beliefs about the “best” approaches to parenting. Further, as it is beyond the scope of this review to provide solutions for addressing the systemic inequities and related psychological challenges that African American mothers face after IPV, we instead make programmatic suggestions that aim to help intervention developers serve this vulnerable population while navigating their current realities. Nonetheless, we recognize that these conceptual decisions may be less informative for those seeking an established IPV parenting intervention that has already been tailored to the parenting context of African American mothers post-IPV. Finally, because African American mothers in the United States were our primary focus due to their overrepresentation in the U.S. criminal justice system and U.S.-based rates of poverty and IPV, it is possible that our recommendations may not be as salient in non-U.S. contexts where systems may operate differently. However, we believe that our proposed roadmap still has utility for other Black groups (e.g., Afro-Caribbean, Afro-Latina), for whom discussions about strengths-based perspectives, specific cultural values, traditions, and racial socialization practices, potential barriers to accessing care, concerns about mandated reporting, use of peer support networks, and the need for resource sharing and psychoeducation are still worthwhile. In these cases, we recommend that program developers also remain attuned to considerations related to immigration status and acculturative processes (Tamis-LeMonda et al., 2008).

Our review and proposed framework are timely and have important implications for ensuring culturally sensitive, nuanced services are made available to this priority population. The practical suggestions and reflections elicited by our illustrative questions serve as a steppingstone for taking the next step in designing, adapting, and implementing culturally sensitive parent–child interventions that fully address the wants and needs of trauma-exposed African American families.

## Author contributions

BC: Conceptualization, Formal analysis, Writing – original draft, Writing – review & editing. MA: Conceptualization, Formal analysis, Project administration, Writing – original draft, Writing – review & editing. QW: Supervision, Writing – review & editing. EJ: Writing – review & editing. CS: Conceptualization, Funding acquisition, Supervision, Writing – review & editing, Investigation.
