# Supplementary material for: Considerations in cultural adaptation of parent–child interventions for African American mothers and children exposed to intimate partner violence
Source: Front Psychol. 2024 Apr 23;15:1295202. doi: 10.3389/fpsyg.2024.1295202 (PMC11074366; doi:10.3389/fpsyg.2024.1295202)
Supplement: Supplementary file 1 [file Table_1.docx]

| **Supplementary Table 1.** Illustrative Questions to Guide Cultural Adaptations of IPV Parent-Child Interventions for African American Mothers. | | | | | | | | |  |
| --- | --- | --- | --- | --- | --- | --- | --- | --- | --- |
|  | **Guiding Principles** |  | **Illustrative Questions** |  |  |  | |  |  |
| Using a strengths-based approach to avoid perpetuation of discrimination against African American women as caregivers | | | 1. Are intervention facilitators engaging with mothers and asking what parenting values are most  salient to them or their community, or have these values already been assumed? | | | | | |  |
|  |  |  | 2. Are intervention facilitators inviting mothers to identify the ways in which they are succeeding  as parents in their own, unique contexts and then using this as an opportunity to enhance their | | | | | |  |
|  |  |  | sense of competence, or is a model of parenting being imposed? | | | | | |  |
|  |  |  | 3. Is an appreciation for the mothers' opinions and experiences being conveyed via frequent check-  ins throughout the intervention and at its conclusion about what worked and did not work, or | | | | | |  |
|  |  |  | are their voices being dismissed? | | | | | |  |
| Integrating racial socialization specific to African American interests and values | | | 1. Does the intervention make space for parents and children to talk about their racial identities and  personal experiences? Do facilitators touch on aspects regarding racial pride, preparation for | | | | | |  |
|  |  |  | racial prejudice, achievement, and racial equality? | | | | | |  |
|  |  |  | 2. Does the intervention explore what it means to be Black in America, especially within the unique  contexts of the participating families (IPV exposure), and how does it acknowledge the historical | | | | | |  |
|  |  |  | and modern-day implications of racism? | | | | | |  |
|  |  |  | 3. To what extent are these conversations being incorporated into the intervention content? Are  these stand-alone modules to be discussed in addition to the "standard" parenting curriculum, | | | | | |  |
|  |  |  | or have the two "contents" been fused together? For group discussions, what will this look like | | | | | |  |
|  |  |  | (e.g., built-in discussion time during each session, in session intervals, as needed)? | | | | | |  |
|  |  |  | 4. What are the mechanisms for transmitting racial socialization messages the intervention  is using? Are both explicit and implicit methods employed? | | |  |  |  |  |
| Embracing African American cultural values and traditions | | |  | | | | | |  |
|  |  |  | 1. Who is included in the caregiving support system of the parents participating in the intervention  (e.g., grandparents, godparents, aunts), and are these members included in discussions? | | | | | |  |
|  |  |  | 2. Are there strong spiritual, religious beliefs that guide the parenting practices of the participants?  How does the intervention ensure that it is respectful of this, and to what extent are parents  encouraged to harness these strongly held beliefs to enhance their parenting?  3. Are program materials sufficiently and appropriately adapted specifically for African American  mothers and children? How are materials evaluated to be culturally relevant and sensitive? Has  feedback been sought from members of the community? | | | | | |  |
|  |  |  | 4. Who is facilitating the intervention- a member of the community with shared experience or an | | | | | |  |
|  |  |  | outside member in a position of power despite having little or no knowledge of the  community? | | | | |  |  |
| Addressing barriers through equity promotion focused on disparities African American women face | | | 1. What factors might prevent a potential participant from participating in the intervention? For | | | | | |  |
|  |  |  | instance, is the program time/ location convenient to them? Do participants require childcare? | | | | | |  |
|  |  |  | Is it expensive for participants to attend? Will participation in the intervention interfere with  participants' work or other obligations (e.g., cooking a meal)? | | | | | |  |
|  |  |  | 2. Given relevant barriers, how can the intervention be modified or adapted to promote equity for  participants? Can transportation be provided? Is it possible to convert some in-person | | | | | |  |
|  |  |  | requirements to virtual? Can relevant technology be provided if the intervention necessitates it? | | | | | |  |
|  |  |  | Can childcare or meals be provided? Can parking, gas, or bus fare be reimbursed? | | | | | |  |
|  |  |  | 3. How can intervention fidelity and adherence be maintained while also promoting  equity? | |  |  |  |  |  |
| Ensuring a safe space and transparency about mandated reporting sensitive to African American disciplinary practices | | | 1. What are the local/state/federal guidelines for mandated reporting? What constitutes physical  abuse? Who needs to be informed when a report is made? | | | | | |  |
|  |  |  | 2. How can I ensure my clients are aware of the requirements I must abide by? At the start of each  session, can I explicitly remind them of what is and is not reportable? | | | | | |  |
|  |  |  | 3. If a CPS report must be made, how can the parent be informed of this process in a manner  that is sensitive and works to maintain the relationship between the parent and intervention  provider? How can the shared priority of the child’s safety be leveraged in the provider-  parent relationship? | | | |  |  |  |
| Adopting peer support networks for African American survivors of IPV | | | 1. Does the intervention take a relational lens that aims to build foundational relationships  between group members, both former and current, and intervention facilitators? Has the  intervention been intentional in bringing together members of the community with shared | | | | | |  |
|  |  |  | cultural and racial experiences?  2. To what extent and how does the intervention aim to cultivate relationships? Have we  established a model for “graduates” of the program to stay connected with each other and  future cohorts? Do we stay in touch with families both formally (e.g., telephone consultations,  booster sessions) and informally? | | | | | |  |
|  |  |  | 3. If the intervention incorporates a group processing element, what will this look like? Do we  intend to integrate sister healing circles? How do we strike a balance between allowing  enough time for group conversations to be healing and empowering while also being  mindful of intervention adherence and curriculum needing to be covered? | | | |  |  |  |
| Providing psychoeducation and resource sharing to address specific gaps identified for African American mothers and children | | | 1. What questions have families asked that are outside the scope of the intervention content? What  information are they seeking or have they conveyed interest in learning about? | | | | | |  |
|  |  |  | 2. What services does this target population need that the intervention can help put them in contact  with? | | | | | |  |
